# Excess labile carbon promotes diazotroph abundance in heat-stressed octocorals

**DOI:** 10.1098/rsos.221268

**Published:** 2023-03-15

**Authors:** Nan Xiang, Achim Meyer, Claudia Pogoreutz, Nils Rädecker, Christian R. Voolstra, Christian Wild, Astrid Gärdes

**Affiliations:** ^1^ Marine Ecology Department, Faculty of Biology and Chemistry, University of Bremen Bremen 28359, Germany; ^2^ Section of Polar Biological Oceanography, Alfred Wegener Institute, Helmholtz Centre for Polar and Marine Research, Bremerhaven 27570, Germany; ^3^ Leibniz Center for Tropical Marine Research (ZMT), Bremen 28359, Germany; ^4^ Department of Biology, University of Konstanz, Konstanz 78457, Germany; ^5^ Laboratory for Biological Geochemistry, School of Architecture, Civil and Environmental Engineering, École Polytechnique Fédérale de Lausanne (EPFL), 1015 Lausanne, Switzerland; ^6^ Hochschule Bremerhaven, Fachbereich 1, An der Karlstadt 8, Bremerhaven 27568, Germany

**Keywords:** coral reefs, global warming, organic eutrophication, symbiosis, N_2_ fixation, octocoral prokaryotes

## Abstract

Nitrogen limitation is the foundation of stable coral-algal symbioses. Diazotrophs, prokaryotes capable of fixing N_2_ into ammonia, support the productivity of corals in oligotrophic waters, but could contribute to the destabilization of holobiont functioning when overstimulated. Recent studies on reef-building corals have shown that labile dissolved organic carbon (DOC) enrichment or heat stress increases diazotroph abundance and activity, thereby increasing nitrogen availability and destabilizing the coral-algal symbiosis. However, the (a)biotic drivers of diazotrophs in octocorals are still poorly understood. We investigated diazotroph abundance (via relative quantification of *nifH* gene copy numbers) in two symbiotic octocorals, the more mixotrophic soft coral *Xenia umbellata* and the more autotrophic gorgonian *Pinnigorgia flava,* under (i) labile DOC enrichment for 21 days, followed by (ii) combined labile DOC enrichment and heat stress for 24 days. Without heat stress, relative diazotroph abundances in *X. umbellata* and *P. flava* were unaffected by DOC enrichment. During heat stress, DOC enrichment (20 and 40 mg glucose l^−1^) increased the relative abundances of diazotrophs by sixfold in *X. umbellata* and fourfold in *P. flava*, compared with their counterparts without excess DOC. Our data suggest that labile DOC enrichment and concomitant heat stress could disrupt the nitrogen limitation in octocorals by stimulating diazotroph proliferation. Ultimately, the disruption of nitrogen cycling may further compromise octocoral fitness by destabilizing symbiotic nutrient cycling. Therefore, improving local wastewater facilities to reduce labile DOC input into vulnerable coastal ecosystems may help octocorals cope with ocean warming.

## 1. Introduction

Coral reefs typically thrive in oligotrophic environments and are among the most diverse and productive ecosystems on Earth [1]. The efficient nutrient recycling between coral holobiont members, i.e. the animal host, photosynthetic dinoflagellates of the family Symbiodiniaceae, and a suite of diverse prokaryotic communities, is the key to enabling this success [2,3]. Indeed, diazotrophs, i.e. prokaryotes capable of converting atmospheric dinitrogen (N_2_) into ammonia, can provide an alternative nitrogen source for the holobiont, sustaining the productivity of corals when environmental nutrient availability is low [4,5]. Diazotrophs are ubiquitous members of reef-building coral and octocoral holobionts with pronounced host-dependent differences in their abundance, activity, and community structure [5–9].

Global and local environmental change threaten to destabilize the functioning of coral holobionts and the reefs they support [10–13]. Importantly, diazotrophs in reef-building corals are susceptible to changing environmental conditions [3,8,9,13–18]. Previous studies have reported that coral bleaching, the stress-induced breakdown of stable holobiont functioning, not only results in the loss of Symbiodiniaceae but also coincides with the opportunistic proliferation of diazotrophs [13,19,20]. The increase in diazotroph-derived nitrogen (DDN) stimulated by heat stress or labile dissolved organic carbon (DOC) enrichment has been suggested to contribute to the disruption of nitrogen limitation, which is typically required for the efficient nutrient exchange between the coral host and Symbiodiniaceae [13,14,19,20].

Similar to reef-building corals, symbiotic octocorals also rely on such intimate metabolic interactions between holobiont members [21,22]. However, octocorals are remarkably resistant to environmental disturbances, such as ocean warming and acidification [23–27]. As a consequence, octocorals are becoming increasingly abundant on degraded coral reefs affected by anthropogenic disturbance [25,27]. Understanding the mechanistic underpinnings of the ecological success of octocorals on degraded reefs could help decipher the processes shaping these emerging novel ecosystems [25,28].

**Figure 1.**
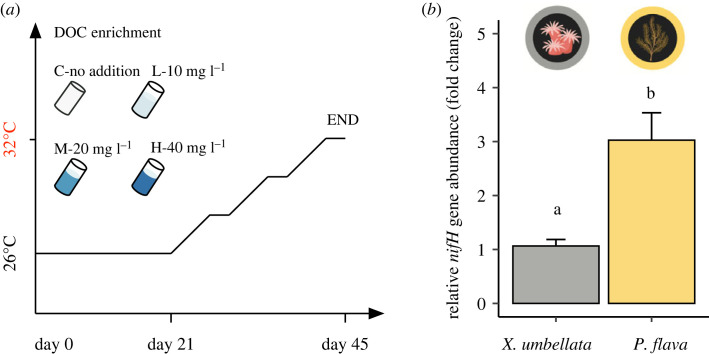
Experimental design of the 45-day aquarium experiment (*a*) and relative diazotroph abundances in the octocorals *Xenia umbellata* and *Pinnigorgia flava* before the start of experiment (*b*). Relative fold change expressed as *nifH* gene copy number in reference to 16S rRNA gene copy numbers and in relation to samples of *X. umbellata* (*n* = 3). Three levels of labile DOC enrichment were achieved by daily glucose administration throughout the experiment. A stepwise increase in temperature was applied from day 21 to day 45.

It is generally recognized that labile DOC enrichment or heat stress can promote the proliferation of diazotrophs in reef-building corals [13–15,18]; however, their impact on diazotrophs in the octocoral holobiont remains unknown. Here, we thus set out to investigate the response of octocoral-associated diazotrophs to labile DOC enrichment and its interaction with heat stress in an aquarium experiment over 45 days (figure 1*a*). Using quantitative polymerase chain reaction (qPCR) of the *nifH* gene which encodes for the iron protein of nitrogenase that catalyses N_2_ fixation as a proxy for N_2_ fixation activity [8], we compared the dynamics of relative diazotroph abundances in two symbiotic octocorals with distinct trophic strategies: the mixotrophic soft coral *Xenia umbellata* (Lamarck, 1816) and the highly autotrophic gorgonian *Pinnigorgia flava* (Nutting, 1910). We addressed the following questions: (i) Do diazotroph abundances differ between octocoral species of distinct trophic strategies under undisturbed conditions? (ii) Does labile DOC enrichment, as a readily available energy source, promote the proliferation of diazotrophs in octocorals? (iii) If so, does the stimulating effect of labile DOC enrichment become more pronounced in heat-stressed octocorals? Thereby, this study is the first to show that heat stress can exacerbate the effects of labile DOC pollution on diazotroph proliferation in octocorals. Notably, this observation may destabilize the nitrogen-limited conditions required for efficient symbiotic nutrient cycling in the octocoral holobiont.

## 2. Materials and methods

### 2.1. Coral husbandry, experimental design and sampling

The soft coral *X. umbellata* mother colonies originated in the northern Red Sea, while the gorgonian *P. flava* mother colonies originated in the Caribbean. Mother colonies of both octocoral species were cultivated for more than 2 years at the indoor aquaria facility (temperature: 26 ± 0.5°C; pH: 7.8 ± 0.2; salinity: 35 ± 3‰) of the Marine Ecology Department at the University of Bremen. This study was a companion experiment to [29] using the same coral fragments and experimental design. In brief, *X. umbellata* colonies were cut into small fragments (1–2 cm side length) and carefully fixed onto calcium carbonate holders (1 × 1 cm) using rubber bands. Likewise, *P. flava* colonies were fragmented into individual branches (3–4 cm in length) and attached to coral holders using aquarium moss coral fix glue (D-D Aquascape Construction Epoxy). A total of 120 *X. umbellata* and *P. flava* fragments each were distributed over 12 aquaria tanks (water volume 50 l each), with 10 fragments of *X. umbellata* and 10 fragments of *P. flava* in each tank. Each tank was equipped with a thermostat (3613 aquarium heater, 75 W 220–240 V, EHEIM, Germany), a pump (CompactOn 300 pump, EHEIM, Germany), a protein skimmer (SkimMarine 100; EHEIM, Germany) and LED lights (Royal Blue-matrix module and Ultra Blue White 1 : 1-matrix module, WALTRAt day time® LED light, Germany) at a 12 : 12 h light : dark cycle at an intensity of 120.8 ± 10.2 µmol quanta m^−2^ s^−1^. Each tank was filled with freshly prepared artificial seawater (salinity: 35.40 ± 0.40‰; pH: 8.20 ± 0.01; temperature: 26 ± 0.4°C) with a daily water renewal rate of 10% to maintain stable water parameters. To avoid excessive biofilm formation, aquaria surfaces were cleaned daily. To avoid confounding effects from additional nutrient uptake via heterotrophy which could have affected the stress response of octocorals, no additional feeding was provided over the course of the experiment.

In the first phase of the experiment (total duration: 21 days), daily dosing of glucose based on a stock solution (d-Glucose, 40 mg ml^−1^) was performed to simulate different levels of DOC loading: 3 mg l^−1^ (control), 10 mg l^−1^ (low), 20 mg l^−1^ (moderate) and 40 mg l^−1^ (high). The glucose concentration levels employed in this experiment were chosen based on previously reported DOC concentrations (in the range from 29 to 233.3 µM) for coral reefs worldwide [30]. Daily measurements of total organic carbon using a TOC-L analyser (Shimadzu, Japan) were performed as a proxy for labile DOC levels, and daily dosings of glucose were accordingly adjusted to achieve the desired levels [29]. In the second phase (total duration of 24 days), aquaria tanks were gradually ramped up to 32°C (close to the thermal threshold of *X. umbellata*) with a 2°C stepwise increase every 8 days [29]. Labile DOC dosing was continued as described above during the second phase. In total, 12 aquaria tanks were evenly distributed among four treatment groups including control, low, moderate and high labile DOC concentrations, with three replicate tanks per treatment group. Coral samples were collected at day 0 (baseline, before labile DOC enrichment), at the end of the first phase (day 21, labile DOC enrichment), and at the end of the second phase (day 45, combined labile DOC enrichment and heat stress). At each sampling time point, coral fragments (three replicates for each treatment) were immediately flash-frozen in liquid nitrogen and subsequently stored at −80°C until further processing.

### 2.2. DNA extraction and *nifH* polymerase chain reaction

Frozen coral samples were ground into powder over liquid nitrogen using a sterilized mortar and pestle. According to the manufacturer's instructions, the powdered samples were directly used for genomic DNA extraction with the Quick-DNA Universal Kit for Solid Tissue (ZYMO RESEARCH, USA). The buffers in this kit are designed to efficiently hydrolyse and remove RNA during the DNA purification procedure. The yield and quality of extracted DNA were assessed by spectrophotometry at 260 and 280 nm using an Infinite 200 PRO (Tecan, Austria) and visually inspected on a 1% (w/v) agarose gel electrophoresis (Biometra Horizon 58, Germany).

A fragment of the *nifH* gene that encodes for the iron protein subunit of nitrogenase was used as a marker to assess diazotroph communities [31]. Eight different primer combinations (electronic supplementary material, figure S1) were evaluated for their ability to amplify fragments of the *nifH* gene in the positive control (see electronic supplementary material for positive control design) and DNA extracted from octocoral holobionts. Based on our evaluation, the primer pair nifH-IGK3 (5′-GCIWTHTAYGGIAARGGIGGIATHGGIAA-3′) and nifH-DVV (5′-ATIGCRAAICCICCRCAIACIACRTC-3′) [31] was selected for the purpose of this study. The PCR reactions consisted of 10 µl of Taq DNA Polymerase Master Mix (VWR, USA), 1 µl GC-enhancers (Applied Biosystems, USA), 2.4 µl of 10 µM forward and 10 µM reverse primer each, 2.2 µl of nuclease-free water and 2 µl DNA template (10 ng µl^−1^) for a total reaction volume of 20 µl. The thermal cycling conditions consisted of an initial denaturation step at 95°C for 2 min, followed by 40 cycles of denaturation at 95°C for 30 s, annealing at 57°C for 30 s and extension at 72°C for 30 s. The specificity of *nifH* amplicons was validated with Sanger sequencing (StarSEQ Mainz, Germany).

### 2.3. *nifH* quantitative polymerase chain reaction

Relative quantification of the *nifH* gene via qPCR was used to assess the relative diazotroph abundance in the octocoral holobiont [8]. Of note, the use of degenerate primers (as used here) may overestimate *nifH* abundance in the sample due to the amplification of *nifH* homologues and the unspecific amplification [32]. However, in the context of the present study where the same organisms were compared under similar conditions, a potential amplification bias is unlikely to drive the treatment-specific effects. The reagent composition and volume for the *nifH* gene amplification were maintained as described above for conventional PCR. The Taq DNA Polymerase Master Mix was replaced by 2X SensiFAST master mix (Bioline, Germany). The *nifH* gene copy numbers (as a proxy of diazotroph abundance) were referenced against the 16S rRNA gene copy numbers (as a reference for total bacterial abundance) amplified by the primer pair Bact-16S_784F (5′-AGGATTAGATACCCTGGTA-3′) and Bact-16S_1061R (5′-CRRCACGAGCTGACGAC-3′) [33], according to the delta-delta Ct method (2^−ΔΔCt^) [34]. The qPCR reactions for the 16S rRNA gene consisted of 1.0 µl forward (10 µM) and reverse primer (10 µM) each, 6.0 µl nuclease-free water, 10.0 µl 2X SensiFAST master mix and 2.0 µl DNA template (10 ng µl^−1^).

The qPCRs were performed on the CFX96 real-time detection system (Bio-Rad, USA), running with an initial polymerase activation at 95°C for 2 min, followed by 40 cycles of denaturation at 95°C for 15 s, primer annealing at 57°C for 30 s (*nifH* gene) or at 60°C for 20 s (16S rRNA gene) and extension at 72°C for 30 s. A final extension was carried out at 72°C for 10 s followed by a melting curve cycle from 65°C to 95°C with an increase of 0.5°C every 5 s. The qPCR efficiency was validated by calibration curves generated using a serial dilution of DNA of *Escherichia* *coli* (reference strain ATCC 25922) targeting the 16S rRNA gene, and DNA of the synthesized partial *nifH* sequence from *Azotobacter vinelandii* DJ targeting the *nifH* gene, respectively. The qPCR efficiency was 90.33% for the *nifH* gene and 92.31% for the 16S rRNA gene.

### 2.4. Statistical analyses

Statistical analyses were conducted in R (v. 4.1.1). Plots were generated using the package ‘ggplot2’ [35]. The qPCR data were log-transformed to meet the assumption of normality as confirmed with the Shapiro–Wilk test using the package ‘nortest’ [36]. The difference in the relative diazotroph abundance between octocoral species at each time point was analysed by a two-way analysis of variance (ANOVA) defining coral species and labile DOC enrichment as factors. Likewise, temporal patterns in the qPCR data were analysed separately for *X. umbellata* and *P. flava* using the two-way ANOVA defining time and labile DOC enrichment as factors, followed by Tukey's honestly significant difference (HSD) as *post hoc* comparisons. All data were presented as mean ± s.e.

## 3. Results

### 3.1. Labile dissolved organic carbon enrichment does not affect relative diazotroph abundance in octocorals at ambient temperature

**Figure 2.**
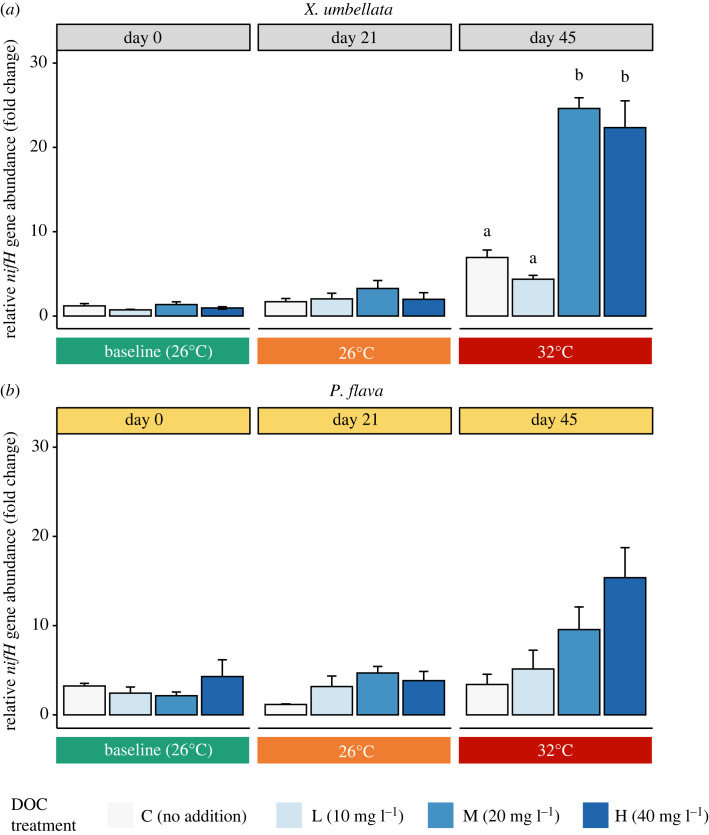
Relative diazotroph abundances in the octocorals *X. umbellata* (*a*) and *P. flava* (*b*) under ambient conditions, labile DOC enrichment and combined labile DOC enrichment and heat stress. Relative fold change in copy numbers of the *nifH* gene referenced to the 16S rRNA gene and in relation to day 0 control samples of *X. umbellata* (*n* = 3). All data are presented as mean ± s.e. (*n* = 3 fragments each). Different letters above bars indicate significant differences between groups of the same time point (Tukey HSD, *p* < 0.05).

Using relative *nifH* gene copy numbers as a proxy for diazotroph abundances, we found that the relative abundance of diazotrophs was significantly higher in *P. flava* than in *X. umbellata* at the beginning of the experiment (figure 1; two-way ANOVA, *F*_1,16_ = 30.07, *p* < 0.001). Twenty-one days of labile DOC enrichment did not alter the relative abundance of diazotrophs in both *X. umbellata* (figure 2; two-way ANOVA, *F*_3,16_ = 1.44, *p* = 0.268) and *P. flava* (figure 2; two-way ANOVA, *F*_3,16_ = 1.72, *p* = 0.202). In *X. umbellata*, low (10 mg l^−1^) and high DOC (40 mg l^−1^) exposure did not affect relative diazotroph abundances, whereas moderate DOC treatment (20 mg l^−1^) increased the relative abundance of diazotrophs by approximately twofold compared with the control (3 mg l^−1^, Tukey HSD, *p* = 0.75). By contrast, the relative diazotroph abundances of *P. flava* exposed to low (10 mg l^−1^, Tukey HSD, *p* = 0.46), moderate (20 mg l^−1^, Tukey HSD, *p* = 0.06) and high DOC (40 mg l^−1^, Tukey HSD, *p* = 0.16) increased non-significantly compared with those from the control group (3 mg l^−1^). As reported in [23,29], holobiont phenotype, photosynthesis, and respiration of both octocoral species were also unaffected by labile DOC enrichment at 26°C.

### 3.2. Labile dissolved organic carbon enrichment boosts relative diazotroph abundance in octocorals during heat stress

Following the gradual increase in seawater temperature to 32°C (i.e. at day 45), the relative abundance of diazotrophs in *X. umbellata* (figure 2; two-way ANOVA, *F*_1,28_ = 106.51, *p* < 0.001) and *P. flava* (figure 2; two-way ANOVA, *F*_1,28_ = 12.30, *p* = 0.003) showed a significant increase compared with day 21 (prior to heat stress). In *X. umbellata*, moderate (20 mg l^−1^) and high labile DOC (40 mg l^−1^) treatments increased the relative abundance of diazotrophs by nearly sixfold compared with the control (3 mg l^−1^) and low labile DOC treatment (10 mg l^−1^) (figure 2; two-way ANOVA, *F*_3,16_ = 9.37, *p* = 0.001). Similarly, a positive effect of labile DOC enrichment on diazotroph abundance was evident in *P. flava*, albeit not significant (figure 2; two-way ANOVA, *F*_3,16_ = 0.79, *p* = 0.520; relative fold change, control: 3.40 ± 1.14; low: 5.14 ± 2.10; moderate: 9.56 ± 2.53; high: 15.37 ± 3.37). Relative diazotroph abundance under high labile DOC treatment increased by nearly fivefold compared with the control under heat stress (Tukey HSD, *p* = 0.06). As stated in [23,29], *X. umbellata* maintained a visibly healthy phenotype with no effects on photosynthesis and respiration rates at different DOC concentrations at 32°C. By contrast, *P. flava* bleached under moderate and high labile DOC enrichments combined with heat stress.

## 4. Discussion

Diazotrophs support the productivity of corals in oligotrophic waters [4,5], but may contribute to the destabilization of holobiont functioning when overstimulated [13,14]. Currently, studies on cnidarian-associated diazotrophs are largely focused on reef-building corals due to their importance as major ecosystem engineers of tropical coral reefs. Here, we provided the first assessment of diazotroph abundance dynamics in two common octocoral species under labile DOC enrichment and its interactions with heat stress over the course of a 45-day aquarium experiment. We found that labile DOC enrichment caused no significant effect on the relative abundance of diazotrophs in the two investigated octocorals at 26°C, but stimulated their abundances by up to sixfold in the soft coral *X. umbellata* and by up to fourfold in the gorgonian *P. flava* at 32°C. Our results suggest that heat stress may stimulate the effect of labile DOC enrichment on the proliferation of diazotrophs in octocorals, possibly destabilizing the nitrogen limitation in octocoral holobionts, as discussed below.

### 4.1. Diazotroph abundances in *Xenia umbellata* and *Pinnigorgia flava* are host species-specific

While host-specific patterns of N_2_ fixation rates have been recorded for octocorals [5,6], molecular characterization is currently missing. Here, we show that the relative abundance of diazotrophs is markedly higher in the autotrophic gorgonian *P. flava* compared with the mixotrophic soft coral *X. umbellata* under undisturbed conditions. This observation aligns with the previously described patterns of diazotroph abundance in reef-building corals [8]. This suggests that the mixotrophic *X. umbellata* exhibits a flexible feeding strategy that allows access to various nutrient sources, thereby potentially rendering it less dependent on diazotrophs as suggested by their lower relative abundances [37]. By contrast, the highly autotrophic *P. flava* may have a stronger dependence on diazotrophs to fulfil the nitrogen requirements for their growth and metabolism [8,38].

Host-specific diazotroph abundances may also be linked to the different morphologies of the two investigated octocorals [38,39]. The *P. flava* holobiont contains an axial skeleton containing calcium carbonate inclusions that may provide a favourable habitat for endolithic bacteria similar to the skeleton of reef-building corals [40–42], which is absent in *X. umbellata*. Of note, endolithic diazotrophs constitute a major component of diazotroph communities in reef-building coral holobionts [6,43]. In addition, *X. umbellata* exhibits a unique pulsation behaviour that provides numerous advantages to the holobiont, including enhanced O_2_ availability and facilitated nutrient supplementation [39,44]. However, as most diazotrophs are particularly sensitive to high oxygen or high inorganic nitrogen levels [45], these benefits may instead constrain the diazotroph abundance and/or activity in this soft coral holobiont. Taken together, our findings suggest that diazotrophs may be common members of the octocoral microbiome, as suggested previously [22], and that patterns of diazotroph abundances align with the heterotrophic capacity and/or morphology of their octocoral host.

### 4.2. Labile dissolved organic carbon stimulates diazotroph proliferation in heat-stressed octocorals

The relative abundances of diazotrophs in both octocoral species were unaffected by labile DOC enrichment at 26°C. This is in stark contrast with previous findings on reef-building corals where 10 mg l^−1^ labile DOC enrichment significantly and rapidly promotes their associated diazotroph abundance and activity, and rapidly results in coral bleaching and a disruption of holobiont nitrogen limitation [13]. Notably, octocorals are commonly found in high abundance on degraded coral reefs threatened by anthropogenic change [25,27]. Recent studies have shown that labile DOC enrichment caused no negative effect on the photosynthetic and respiration rates of octocorals [23], and a similar phenomenon was observed in the stress-tolerant symbiotic upside-down jellyfish *Cassiopea* [46]. As such, octocoral holobionts may be better adapted to cope with environmental labile DOC enrichment compared with reef-building coral holobionts. At this point, it remains unclear why relative abundances of diazotrophs in octocorals remain stable during labile DOC enrichment. Yet, this finding may in part explain the success of octocorals in disturbed coral reef environments [25].

During 32°C heat stress, moderate (20 mg glucose l^−1^) and high (40 mg glucose l^−1^) labile DOC enrichment significantly promoted the relative abundances of *nifH* gene copy numbers in two octocoral species: the soft coral *X. umbellata* and the gorgonian *P. flava*. Such increases in *nifH* abundance have previously been linked to concomitant increases in *nifH* gene expression and N_2_ fixation rates and could therefore be used as a proxy of diazotroph activity [8]. Further, increases in diazotroph abundance and activity have been linked to increases in nitrogen availability in the holobiont, contributing to bleaching and the symbiotic breakdown of reef-building corals [13]. In this light, similar mechanisms may be present in symbiotic octocoral holobionts. At this point, the role of diazotrophs in determining the species-specific bleaching response in our investigated octocorals remains yet to be determined. Nevertheless, the observed increase in diazotroph abundance in both octocoral species during labile DOC enrichment and heat stress suggests that the pattern of diazotroph dynamics may be similar across cnidarian holobionts with different trophic strategies and lifestyles. However, the effects of stimulated N_2_ fixation on holobiont functioning probably depend on the nutritional status of all holobiont members [13,14]. Taken together, our findings suggest that octocorals exhibit remarkably higher resistance to labile DOC enrichment in comparison with reef-building corals. However, in future ocean warming scenarios, labile DOC enrichment may still be a driver of diazotroph proliferation and thereby increase nitrogen availability in the octocoral holobiont.

## 5. Conclusion

Octocorals constitute an important component of benthic reef communities [21] and are likely to become one of the dominant functional groups on degraded coral reefs of the future [28]. Here we show that the relative abundance of diazotrophs in octocorals appears to be less affected by labile DOC enrichment compared with those in reef-building coral holobionts. Concomitant with heat stress, labile DOC enrichment could significantly stimulate the abundance of diazotrophs in octocorals. As a result, labile DOC enrichment and heat stress may jointly disrupt the nitrogen limitation in octocorals, contributing to the destabilization of holobiont nutrient cycling. As DOC levels comparable to or in excess of what was employed in the 'high' DOC treatment of this study have indeed been reported on different coral reefs, elevated DOC loading is a realistic threat that may undermine the fitness of octocorals locally [47]. Consequently, reducing labile DOC loading from local anthropogenic activities is imperative to assisting octocorals and other photosymbiotic cnidarians in coping with the effects of ocean warming.

## Data Availability

The supporting information is provided in the electronic supplementary material. Raw data and R code used for data visualization and statistics are provided in the Dryad Digital Repository: https://doi.org/10.5061/dryad.mw6m9060q [48]. The data are provided in the electronic supplementary material [49].

## References

[1] Darwin C. 1889 The structure and distribution of coral reefs. London, UK: Smith, Elder & Co. (10.1525/9780520327337)

[2] Muscatine L, Porter JW. 1977 Reef corals: mutualistic symbioses adapted to nutrient-poor environments. Bioscience **27**, 454-460. (10.2307/1297526)

[3] Rädecker N, Pogoreutz C, Voolstra CR, Wiedenmann J, Wild C. 2015 Nitrogen cycling in corals: the key to understanding holobiont functioning? Trends Microbiol. **23**, 490-497. (10.1016/j.tim.2015.03.008) 25868684

[4] Cardini U, Bednarz VN, Naumann MS, van Hoytema N, Rix L, Foster RA, Al-Rshaidat MM, Wild C. 2015 Functional significance of dinitrogen fixation in sustaining coral productivity under oligotrophic conditions. Proc. R. Soc. B **282**, 20152257. (10.1098/rspb.2015.2257) PMC4650168 26511052

[5] Bednarz V, Cardini U, Van Hoytema N, Al-Rshaidat M, Wild C. 2015 Seasonal variation in dinitrogen fixation and oxygen fluxes associated with two dominant zooxanthellate soft corals from the northern Red Sea. Mar. Ecol. Prog. **519**, 141-152. (10.3354/meps11091)

[6] Shashar N, Feldstein T, Cohen Y, Loya Y. 1994 Nitrogen fixation (acetylene reduction) on a coral reef. Coral Reefs **13**, 171-174. (10.1007/bf00301195)

[7] Lema KA, Willis BL, Bourne DG. 2012 Corals form characteristic associations with symbiotic nitrogen-fixing bacteria. Appl. Environ. Microbiol. **78**, 3136-3144. (10.1128/AEM.07800-11) 22344646 PMC3346485

[8] Pogoreutz C, Rädecker N, Cárdenas A, Gärdes A, Wild C, Voolstra CR. 2017 Nitrogen fixation aligns with *nifH* abundance and expression in two coral trophic functional groups. Front. Microbiol. **8**, 1187. (10.3389/fmicb.2017.01187) 28702013 PMC5487474

[9] Geissler L, Meunier V, Rädecker N, Perna G, Rodolfo-Metalpa R, Houlbrèque F, Voolstra CR. 2021 Highly variable and non-complex diazotroph communities in corals from ambient and high CO_2_ environments. Front. Mar. Sci. **8**, 754682. (10.3389/fmars.2021.754682)

[10] Hughes TP, et al. 2017 Global warming and recurrent mass bleaching of corals. Nature **543**, 373-377. (10.1038/nature21707) 28300113

[11] Hughes TP, et al. 2003 Climate change, human impacts, and the resilience of coral reefs. Science **301**, 929-933. (10.1126/science.1085046) 12920289

[12] Vega Thurber RL, Burkepile DE, Fuchs C, Shantz AA, McMinds R, Zaneveld JR. 2014 Chronic nutrient enrichment increases prevalence and severity of coral disease and bleaching. Glob. Chang. Biol. **20**, 544-554. (10.1111/gcb.12450) 24277207

[13] Pogoreutz C, Rädecker N, Cárdenas A, Gärdes A, Voolstra CR, Wild C. 2017 Sugar enrichment provides evidence for a role of nitrogen fixation in coral bleaching. Glob. Chang. Biol. **23**, 3838-3848. (10.1111/gcb.13695) 28429531

[14] Rädecker N, et al. 2021 Heat stress reduces the contribution of diazotrophs to coral holobiont nitrogen cycling. ISME J. **16**, 1110-1118. (10.1038/s41396-021-01158-8) 34857934 PMC8941099

[15] Santos HF, Carmo FL, Duarte G, Dini-Andreote F, Castro CB, Rosado AS, Van Elsas JD, Peixoto RS. 2014 Climate change affects key nitrogen-fixing bacterial populations on coral reefs. ISME J. **8**, 2272-2279. (10.1038/ismej.2014.70) 24830827 PMC4992079

[16] Meunier V, et al. 2021 Microbes support enhanced nitrogen requirements of coral holobionts in a high CO_2_ environment. Mol. Ecol. **30**, 5888-5899. (10.1111/mec.16163) 34473860

[17] El-Khaled YC, Roth F, Rädecker N, Tilstra A, Karcher DB, Kürten B, Jones BH, Voolstra CR, Wild C. 2021 Nitrogen fixation and denitrification activity differ between coral- and algae-dominated Red Sea reefs. Sci. Rep. **11**, 1-15. (10.1038/s41598-021-90204-8) 34083565 PMC8175748

[18] El-Khaled YC, Nafeh R, Roth F, Rädecker N, Karcher DB, Jones BH, Voolstra CR, Wild C. 2021 High plasticity of nitrogen fixation and denitrification of common coral reef substrates in response to nitrate availability. Mar. Pollut. Bull. **168**, 112430. (10.1016/j.marpolbul.2021.112430) 34000709

[19] Bednarz VN, van de Water JA, Rabouille S, Maguer JF, Grover R, Ferrier-Pagès C. 2019 Diazotrophic community and associated dinitrogen fixation within the temperate coral *Oculina patagonica*. Environ. Microbiol. **21**, 480-495. (10.1111/1462-2920.14480) 30452101

[20] Rädecker N et al. 2021 Heat stress destabilizes symbiotic nutrient cycling in corals. Proc. Natl Acad. Sci. USA **118**, e2022653118. (10.1073/pnas.2022653118) 33500354 PMC7865147

[21] Schubert N, Brown D, Rossi S. 2016 Symbiotic versus non-symbiotic octocorals: physiological and ecological implications. In Marine animal forests (eds S Rossi, L Bramanti, A Gori, C Orejas Saco del Valle), pp. 887-918. Berlin, Germany: Springer. (10.1007/978-3-319-17001-5_54-1)

[22] Van De Water JAJM, Allemand D, Ferrier-Pagès C. 2018 Host-microbe interactions in octocoral holobionts - recent advances and perspectives. Microbiome **6**, 1-28. (10.1186/s40168-018-0431-6) 29609655 PMC5880021

[23] Simancas-Giraldo SM, Xiang N, Kennedy MM, Nafeh R, Zelli E, Wild C. 2021 Photosynthesis and respiration of the soft coral *Xenia umbellata* respond to warming but not to organic carbon eutrophication. PeerJ **9**, e11663. (10.7717/peerj.11663) 34395065 PMC8323596

[24] Vollstedt S, Xiang N, Simancas-Giraldo SM, Wild C. 2020 Organic eutrophication increases resistance of the pulsating soft coral *Xenia umbellata* to warming. PeerJ **8**, e9182. (10.7717/peerj.9182) 32607278 PMC7316076

[25] Norström A, Nyström M, Lokrantz J, Folke C. 2009 Alternative states on coral reefs: beyond coral–macroalgal phase shifts. Mar. Ecol. Prog. Ser. **376**, 295-306. (10.3354/meps07815)

[26] Lopes AR, Faleiro F, Rosa IC, Pimentel MS, Trubenbach K, Repolho T, Diniz M, Rosa R. 2018 Physiological resilience of a temperate soft coral to ocean warming and acidification. Cell Stress Chaperones **23**, 1093-1100. (10.1007/s12192-018-0919-9) 29948929 PMC6111073

[27] Inoue S, Kayanne H, Yamamoto S, Kurihara H. 2013 Spatial community shift from hard to soft corals in acidified water. Nat. Clim. **3**, 683-687. (10.1038/nclimate1855)

[28] Hoegh-Guldberg O. 1999 Climate change, coral bleaching and the future of the world's coral reefs. Mar. Freshw. Res. **50**, 839. (10.1071/mf99078)

[29] Xiang N, Hassenrück C, Pogoreutz C, Rädecker N, Simancas-Giraldo SM, Voolstra CR, Wild C, Gärdes A. 2022 Contrasting microbiome dynamics of putative denitrifying bacteria in two octocoral species exposed to dissolved organic carbon (DOC) and warming. Appl. Environ. Microbiol. **88**, e01886-21. (10.1128/AEM.01886-21) 34788073 PMC8788706

[30] Haas AF, et al. 2016 Global microbialization of coral reefs. Nat. Microbiol. **1**, 16042. (10.1038/nmicrobiol.2016.42) 27572833

[31] Gaby JC, Buckley DH. 2012 A comprehensive evaluation of PCR primers to amplify the *nifH* gene of nitrogenase. PLoS ONE **7**, e42149. (10.1371/journal.pone.0042149) 22848735 PMC3405036

[32] Gaby JC, Buckley DH. 2017 The use of degenerate primers in qPCR analysis of functional genes can cause dramatic quantification bias as revealed by investigation of *nifH* primer performance. Microb. Ecol. **74**, 701-708. (10.1007/s00248-017-0968-0) 28389727

[33] Andersson AF, Lindberg M, Jakobsson H, Bäckhed F, Nyrén P, Engstrand L. 2008 Comparative analysis of human gut microbiota by barcoded pyrosequencing. PLoS ONE **3**, e2836. (10.1371/journal.pone.0002836) 18665274 PMC2475661

[34] Livak KJ, Schmittgen TD. 2001 Analysis of relative gene expression data using real-time quantitative PCR and the 2−*ΔΔ*CT method. Methods **25**, 402-408. (10.1006/meth.2001.1262) 11846609

[35] Wickham H. 2011 ggplot2. Wiley Interdisc. Rev. **3**, 180-185. (10.1002/wics.147)

[36] Gross J, Ligges U, Ligges MU. 2015 Package ‘nortest’: tests for normality. R package version 1.0-4. See https://cran.r-project.org/web/packages/nortest/index.html.

[37] Fabricius K, Klumpp D. 1995 Widespread mixotrophy in reef-inhabiting soft corals: the influence of depth, and colony expansion and contraction on photosynthesis. Mar. Ecol. Prog. **125**, 195-204. (10.3354/meps125195)

[38] Baker DM, Freeman CJ, Knowlton N, Thacker RW, Kim K, Fogel ML. 2015 Productivity links morphology, symbiont specificity and bleaching in the evolution of Caribbean octocoral symbioses. ISME J. **9**, 2620-2629. (10.1038/ismej.2015.71) 25989369 PMC4817624

[39] Kremien M, Shavit U, Mass T, Genin A. 2013 Benefit of pulsation in soft corals. Proc. Natl Acad. Sci. USA **110**, 8978-8983. (10.1073/pnas.1301826110) 23610420 PMC3670383

[40] Boller M, Swain T, Lasker H. 2002 Skeletal morphology and material properties of a fragmenting gorgonian coral. Mar. Ecol. Prog. **228**, 131-141. (10.3354/meps228131)

[41] Goldberg WM. 1976 Comparative study of the chemistry and structure of gorgonian and antipatharian coral skeletons. Mar. Biol. **35**, 253-267. (10.1007/bf00396873)

[42] Pernice M, Raina JB, Rädecker N, Cárdenas A, Pogoreutz C, Voolstra CR. 2020 Down to the bone: the role of overlooked endolithic microbiomes in reef coral health. ISME J. **14**, 325-334. (10.1038/s41396-019-0548-z) 31690886 PMC6976677

[43] Yang SH, Lee STM, Huang CR, Tseng CH, Chiang PW, Chen CP, Chen HJ, Tang SL. 2016 Prevalence of potential nitrogen-fixing, green sulfur bacteria in the skeleton of reef-building coral *Isopora palifera*. Limnol. Oceanogr. **61**, 1078-1086. (10.1002/lno.10277)

[44] Wild C, Naumann MS. 2013 Effect of active water movement on energy and nutrient acquisition in coral reef-associated benthic organisms. Proc. Natl Acad. Sci. USA **110**, 8767-8768. (10.1073/pnas.1306839110) 23671104 PMC3670397

[45] Ormeño-Orrillo E, Hungria M, Martinez-Romero E. 2013 Dinitrogen-fixing prokaryotes. In The prokaryotes (eds E Rosenberg, EF DeLong, S Lory, E Stackebrandt, F Thompson), pp. 427-451. Berlin, Germany: Springer. (10.1007/978-3-642-30141-4_72)

[46] Rädecker N, Pogoreutz C, Wild C, Voolstra CR. 2017 Stimulated respiration and net photosynthesis in *Cassiopeia* sp. during glucose enrichment suggests *in hospite* CO_2_ limitation of algal endosymbionts. Front. Mar. Sci. **4**, 267. (10.3389/fmars.2017.00267)

[47] Meirelles PM, et al. 2018 Metagenomics of coral reefs under phase shift and high hydrodynamics. Front. Microbiol. **9**, 2203. (10.3389/fmicb.2018.02203) 30337906 PMC6180206

[48] Xiang N, Meyer A, Pogoreutz C, Rädecker N, Voolstra CR, Wild C, Gärdes A. 2023 Data from: Excess labile carbon promotes diazotroph abundance in heat-stressed octocorals. *Dryad Digital Repository*. (10.5061/dryad.mw6m9060q) PMC10014249 36938541

[49] Xiang N, Meyer A, Pogoreutz C, Rädecker N, Voolstra CR, Wild C, Gärdes A. 2023 Excess labile carbon promotes diazotroph abundance in heat-stressed octocorals. *Figshare*. (10.6084/m9.figshare.c.6456207) PMC10014249 36938541

